# 3-(4-Hy­droxy­phen­yl)-1,5-bis­(pyridin-2-yl)pentane-1,5-dione

**DOI:** 10.1107/S1600536813025518

**Published:** 2013-09-21

**Authors:** Lixia Pan, Huaduan Shi, Zhen Ma

**Affiliations:** a State Key Laboratory of Non-Food Biomass and Enzyme Technology, National Engineering Research Center for Non-Food Biorefinery, Guangxi Key Laboratory of Biorefinery, Guangxi Academy of Sciences, Nanning, Guangxi 530007, People’s Republic of China; b School of Chemistry and Chemical Engeneering, Guangxi University, Nanning, Guangxi 530004, People’s Republic of China

## Abstract

In the title mol­ecule, C_21_H_18_N_2_O_3_, the pyridine rings make a dihedral angle of 13.1 (1)°. The phenyl ring is approximately perpendicular to both of them, forming dihedral angles of 87.4 (1)and 81.9 (1)°. In the crystal, pairs of O—H⋯N hydrogen bonds link the mol­ecules into centrosymmetric dimers. Additional C—H⋯O, π–π [centroid–centroid distance = 3.971 (2) Å] and C—H⋯π inter­actions consolidate the dimers into a three-dimensional network.

## Related literature

For the synthesis of the title compound, see: Constable *et al.* (1990 ▶, 1998 ▶); He *et al.* (2006 ▶). For the syntheses of terpyridine compounds and their properties and applications, see: Ma *et al.* (2009 ▶, 2010 ▶, 2012 ▶, 2013 ▶). For standard bond lengths, see: Allen *et al.* (1987 ▶).
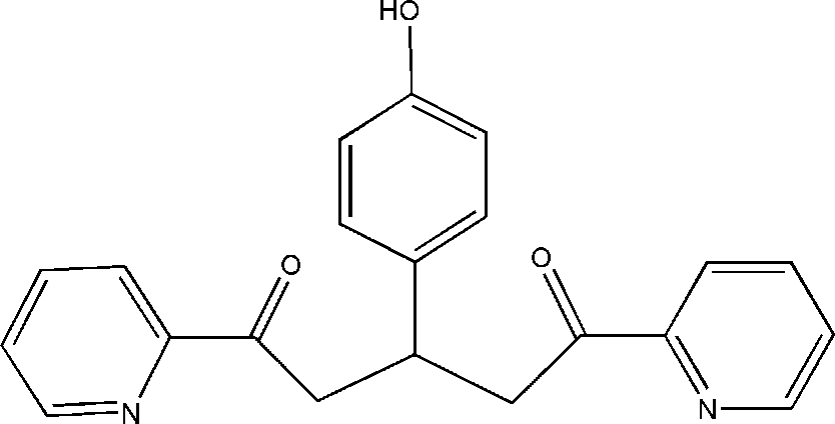


## Experimental

### 

#### Crystal data

C_21_H_18_N_2_O_3_
*M* *_r_* = 346.37
Triclinic, 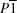

*a* = 8.4392 (6) Å
*b* = 10.6683 (7) Å
*c* = 11.0755 (8) Å
α = 100.623 (6)°
β = 103.867 (6)°
γ = 110.550 (6)°
*V* = 866.05 (10) Å^3^
*Z* = 2
Mo *K*α radiation
μ = 0.09 mm^−1^
*T* = 298 K
0.39 × 0.38 × 0.22 mm

#### Data collection

Agilent SuperNova (Dual, Cu at zero, Atlas) diffractometer
Absorption correction: multi-scan (*CrysAlis PRO*, Agilent, 2012 ▶) *T*_min_ = 0.813, *T*_max_ = 1.000
6339 measured reflections
3544 independent reflections
2661 reflections with *I* > 2σ(*I*)
*R*_int_ = 0.017

#### Refinement

*R*[*F* ^2^ > 2σ(*F* ^2^)] = 0.043
*wR*(*F* ^2^) = 0.119
*S* = 1.03
3544 reflections
308 parameters
All H-atom parameters refined
Δρ_max_ = 0.18 e Å^−3^
Δρ_min_ = −0.14 e Å^−3^

### 

Data collection: *CrysAlis PRO* (Agilent, 2012 ▶); cell refinement: *CrysAlis PRO*; data reduction: *CrysAlis PRO*; program(s) used to solve structure: *SHELXS97* (Sheldrick, 2008 ▶); program(s) used to refine structure: *SHELXL97* (Sheldrick, 2008 ▶); molecular graphics: *Mercury* (Macrae *et al.*, 2008 ▶); software used to prepare material for publication: *SHELXL97* and *PLATON* (Spek, 2009 ▶).

## Supplementary Material

Crystal structure: contains datablock(s) I, C21H18N2O3. DOI: 10.1107/S1600536813025518/ld2113sup1.cif

Structure factors: contains datablock(s) I. DOI: 10.1107/S1600536813025518/ld2113Isup2.hkl

Supplementary material file. DOI: 10.1107/S1600536813025518/ld2113Isup3.cml

Additional supplementary materials:  crystallographic information; 3D view; checkCIF report

## Figures and Tables

**Table 1:** Hydrogen-bond geometry (Å, °) *Cg*3 is the centroid of the C16–C21 ring.

*D*—H⋯*A*	*D*—H	H⋯*A*	*D*⋯*A*	*D*—H⋯*A*
O3—H03*A*⋯N2^i^	0.93 (2)	2.00 (2)	2.8940 (19)	160 (2)
C12—H12*A*⋯O2^ii^	0.96 (2)	2.48 (2)	3.312 (3)	145 (2)
C4a—H4a⋯*Cg*3^iii^	0.99 (2)	0.98 (2)	3.825 (2)	144 (2)

Symmetry codes: (i) 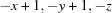; (ii) 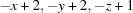; (iii) 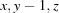.

**Fig. 1.**
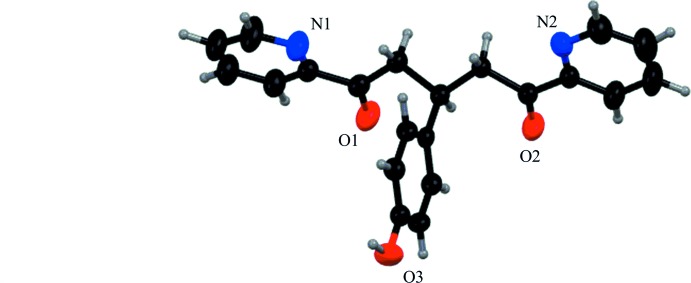
The molecular structure of the title compound with the atom numbering scheme. Displacement ellipsoids are drawn at the 50% probability level. H atoms are presented as small spheres of arbitrary radius.

**Fig. 2.**
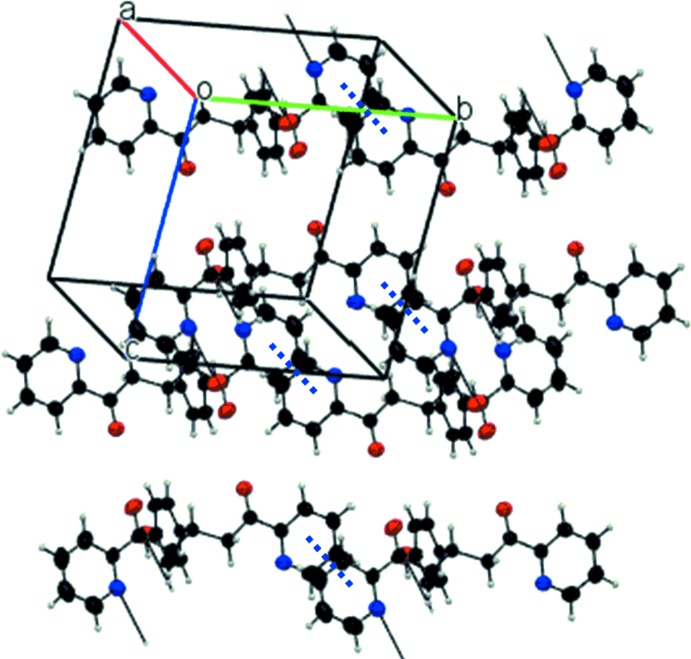
A view of the crystal packing to show the formation of centrosymmetric H-bonded dimers by the help of the hydrogen bonds and the π···π interactions between the pyridyl groups of the compound. The thin dashed lines are used to show the hydrogen bonds. The blue dotted lines are used to show π···π interactions between the pyridyl groups of the compound

**Fig. 3.**
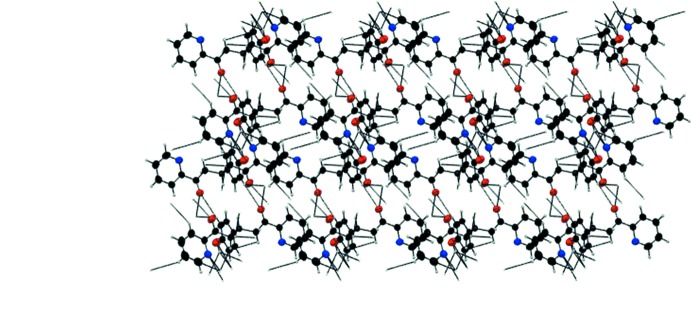
A view of the crystal packing along the a axis to show the three-dimensional network.

## supplementary crystallographic information

### 1. Comment

Metal terpyridene complexes is a topic of major current interest because they show very interesting properties, such as photoluminescence, catalytic and antibiological activities (Ma, Xing *et al.* 2009; Ma, Cao *et al.* 2010; Ma, Liang *et al.* 2012; Ma, Lu *et al.* 2013). Hence, the aim of our current work was to prepare a series of precursors to produce terpyridine ligands, investigate their coordination behavior toward metal ions and study their applications (Constable, Lewis *et al.* 1990; Constable, Neuburger *et al.* 1998). Here, we report the structure of a precursor compound for terpyridine synthesis, which was obtained by reaction of 4-hydroxybenzaldehyde with 2-acetylpyridine in a mixed water/ethanol solution of NaOH, and its structure was determined by X-ray crystal analysis.

The molecular structure is shown in Fig. 1. The average bond length C=O for two carbonyls is 1.1212 Å. Other averages are 1.336 Å for N-C bonds, 1.371 Å for C-C bonds of the pyridyl groups and 1.384 Å for C-C bonds of the aryl group. All bond lengths are within normal ranges (Allen *et al.*, 1987). The two pyridyl groups are not parallel, with a dihedral angle of 13.14 (10) °. The plane of aromatic ring with the hydroxyl group (with an r.m.s. deviation of 0.0041 Å) is approximately perpendicular to those of the two pyridyl groups, forming two dihedral angles of 87.36 (5) and 81.90 (6)°, respectively.

Each molecule forms hydrogen bonds (Table 1) involving its hydroxy group and a nitrogen pyridyl atom (N2^ii^) of a neighboring molecule [symmetry code: (ii) 1-*x*, 1-*y*, -*z*]) and also an H-bond between C(12)-H group and a carbonyl oxygen (O2^iii^) of a neighboring molecule [symmetry code: (iii) 2-*x*, 2-*y*, 1-*z*] (Table 1). These hydrogen bonds account for the formation of centrosymmetric dimers (see Fig 2). The structure also has one intermolecular π···π interaction with a distance of 3.971 Å between two neighboring pyridyl groups (see Fig 2)[Cg1 and Cg2(iv) or Cg2 and Cg1(v); Cg2 and Cg1 are the two centroids of the two six membered pyridyl rings of C1-C5-N1 or C11-C15-N2, symmetry code: (iv) -1+*x*, -1+*y*, *z*; (v) 1+*x*, 1+*y*, *z*]. Further structural stabilization is provided by an intermolecular C—H···π interaction between C(4)-H and its neighboring aryl group Cg3 (Fig 2) [H..Cg 2.98 Å; Cg(3) is the centroid of the six membered aromatic ring C16^i^-C21^i^, symmetry code: (i) *x*, -1+*y*-1, *z*]. These π···π and C—H···π interactions help to consolidate the H-bonded dimers into a three-dimensional network (Fig 3).

### 2. Experimental

The title compound was obtained by reaction of 4-hydroxybenzaldehyde with 2-acetylpyridine in a 1.5 *M* NaOH mixed aqueous/ethanol solution according to a reported procedure (Constable, *et al.* 1990). In a 250 cm^3^ flask fitted with a funnel, 4-hydroxybenzaldehyde (5.5 g, 45 m*M*) and 40 mL of the 1.5 M NaOH aqueous solution were mixed in 60 cm^3^ of ethanol. To this solution was added dropwise a stoichiometric quantity of 2-acetylpyridine (10 mL, 89 m*M*) for a period of half an hour with stirring. The mixture was then stirred for 24 h at room temperature. A white solid formed was obtained by filtration and being washed with two times with distilled water (yield 70 %). The product (25 mg) and distilled water (20 mL) were sealed in a 25-mL stainless steel reactor with Teflon liner and heated at 393 K for 1 d. Colourless crystals were obtained, which were suitable for X-ray characterization.

### 3. Refinement

All H atoms were positioned from a Difference Fourier series and refined.

### Crystal data

**Table:**

C_21_H_18_N_2_O_3_	*Z* = 2
*M**_r_* = 346.37	*F*(000) = 364
Triclinic, *P*1	*D*_x_ = 1.328 Mg m^−^^3^
Hall symbol: -P 1	Mo *K*α radiation, λ = 0.71073 Å
*a* = 8.4392 (6) Å	Cell parameters from 6339 reflections
*b* = 10.6683 (7) Å	θ = 3.2–26.4°
*c* = 11.0755 (8) Å	µ = 0.09 mm^−^^1^
α = 100.623 (6)°	*T* = 298 K
β = 103.867 (6)°	Prism, colourless
γ = 110.550 (6)°	0.39 × 0.38 × 0.22 mm
*V* = 866.05 (10) Å^3^	

### Data collection

**Table:**

Agilent SuperNova (Dual, Cu at zero, Atlas) diffractometer	3544 independent reflections
Radiation source: SuperNova (Mo) X-ray Source	2661 reflections with *I* > 2σ(*I*)
Mirror monochromator	*R*_int_ = 0.017
Detector resolution: 0 pixels mm^-1^	θ_max_ = 26.4°, θ_min_ = 3.2°
ω scans	*h* = −10→9
Absorption correction: multi-scan (*CrysAlis PRO*, Agilent, 2012)	*k* = −13→13
*T*_min_ = 0.813, *T*_max_ = 1.000	*l* = −13→11
6339 measured reflections	

### Refinement

**Table:**

Refinement on *F*^2^	Secondary atom site location: difference Fourier map
Least-squares matrix: full	Hydrogen site location: inferred from neighbouring sites
*R*[*F*^2^ > 2σ(*F*^2^)] = 0.043	All H-atom parameters refined
*wR*(*F*^2^) = 0.119	*w* = 1/[σ^2^(*F*_o_^2^) + (0.0498*P*)^2^ + 0.1436*P*] where *P* = (*F*_o_^2^ + 2*F*_c_^2^)/3
*S* = 1.03	(Δ/σ)_max_ < 0.001
3544 reflections	Δρ_max_ = 0.18 e Å^−^^3^
308 parameters	Δρ_min_ = −0.14 e Å^−^^3^
0 restraints	Extinction correction: *SHELXL*, Fc^*^=kFc[1+0.001xFc^2^λ^3^/sin(2θ)]^-1/4^
Primary atom site location: structure-invariant direct methods	Extinction coefficient: 0.018 (3)

### Special details

**Table:**

Geometry. All esds (except the esd in the dihedral angle between two l.s. planes) are estimated using the full covariance matrix. The cell esds are taken into account individually in the estimation of esds in distances, angles and torsion angles; correlations between esds in cell parameters are only used when they are defined by crystal symmetry. An approximate (isotropic) treatment of cell esds is used for estimating esds involving l.s. planes.
Refinement. Refinement of F^2^ against ALL reflections. The weighted R-factor wR and goodness of fit S are based on F^2^, conventional R-factors R are based on F, with F set to zero for negative F^2^. The threshold expression of F^2^ > 2sigma(F^2^) is used only for calculating R-factors(gt) etc. and is not relevant to the choice of reflections for refinement. R-factors based on F^2^ are statistically about twice as large as those based on F, and R- factors based on ALL data will be even larger.

### Fractional atomic coordinates and isotropic or equivalent isotropic displacement parameters (Å^2^)

**Table:**

	*x*	*y*	*z*	*U*_iso_*/*U*_eq_	
O1	0.71359 (19)	0.30614 (12)	0.46604 (11)	0.0618 (4)	
O2	0.91770 (19)	0.78344 (12)	0.42074 (12)	0.0667 (4)	
O3	0.08708 (17)	0.40522 (14)	0.08964 (13)	0.0592 (3)	
H03A	0.044 (3)	0.351 (3)	0.003 (2)	0.100 (8)*	
N1	0.6613 (2)	0.06026 (14)	0.18572 (13)	0.0603 (4)	
N2	1.06938 (19)	0.81798 (14)	0.15318 (13)	0.0493 (4)	
C1	0.6711 (2)	0.10690 (15)	0.30835 (14)	0.0409 (4)	
C2	0.6203 (3)	0.01932 (17)	0.38203 (17)	0.0545 (5)	
H2A	0.632 (2)	0.0607 (19)	0.4701 (18)	0.060 (5)*	
C3	0.5570 (3)	−0.12308 (18)	0.32835 (19)	0.0639 (5)	
H3A	0.524 (3)	−0.184 (2)	0.3782 (19)	0.078 (6)*	
C4	0.5449 (3)	−0.17259 (19)	0.20295 (19)	0.0680 (6)	
H4A	0.501 (3)	−0.273 (2)	0.159 (2)	0.083 (6)*	
C5	0.5963 (4)	−0.07886 (19)	0.1355 (2)	0.0791 (7)	
H5A	0.586 (3)	−0.110 (2)	0.044 (2)	0.092 (7)*	
C6	0.7379 (2)	0.26254 (15)	0.36597 (14)	0.0414 (4)	
C7	0.8359 (2)	0.35918 (16)	0.29975 (17)	0.0432 (4)	
H7A	0.966 (3)	0.3793 (18)	0.3406 (17)	0.060 (5)*	
H7B	0.798 (2)	0.3108 (17)	0.2046 (16)	0.049 (4)*	
C8	0.8128 (2)	0.49738 (14)	0.32322 (14)	0.0378 (3)	
H8A	0.847 (2)	0.5387 (15)	0.4188 (15)	0.040 (4)*	
C9	0.9371 (2)	0.60196 (15)	0.27268 (16)	0.0409 (4)	
H9B	1.059 (2)	0.6076 (17)	0.2993 (15)	0.052 (5)*	
H9A	0.897 (2)	0.5730 (17)	0.1737 (17)	0.052 (5)*	
C10	0.9588 (2)	0.74944 (15)	0.32631 (15)	0.0426 (4)	
C11	1.0436 (2)	0.85928 (15)	0.26587 (15)	0.0428 (4)	
C12	1.0940 (3)	0.99903 (18)	0.3296 (2)	0.0623 (5)	
H12A	1.072 (3)	1.023 (2)	0.410 (2)	0.075 (6)*	
C13	1.1764 (3)	1.0996 (2)	0.2766 (2)	0.0798 (7)	
H13A	1.215 (3)	1.200 (3)	0.322 (2)	0.108 (8)*	
C14	1.2058 (3)	1.0592 (2)	0.1633 (2)	0.0808 (7)	
H14A	1.263 (3)	1.123 (3)	0.122 (2)	0.100 (8)*	
C15	1.1504 (3)	0.9187 (2)	0.1043 (2)	0.0665 (5)	
H15A	1.167 (3)	0.886 (2)	0.0226 (19)	0.068 (6)*	
C16	0.6193 (2)	0.47157 (13)	0.26132 (13)	0.0352 (3)	
C17	0.5210 (2)	0.51095 (15)	0.33320 (15)	0.0404 (4)	
H17A	0.582 (2)	0.5549 (16)	0.4262 (16)	0.047 (4)*	
C18	0.3451 (2)	0.48794 (16)	0.27609 (15)	0.0439 (4)	
H18A	0.281 (2)	0.5150 (18)	0.3262 (16)	0.055 (5)*	
C19	0.2610 (2)	0.42459 (15)	0.14348 (15)	0.0412 (4)	
C20	0.3557 (2)	0.38460 (16)	0.06964 (15)	0.0431 (4)	
H20A	0.297 (2)	0.3412 (18)	−0.0231 (17)	0.056 (5)*	
C21	0.5317 (2)	0.40803 (15)	0.12846 (14)	0.0412 (4)	
H21A	0.598 (2)	0.3788 (17)	0.0732 (16)	0.052 (4)*	

### Atomic displacement parameters (Å^2^)

**Table:**

	*U*^11^	*U*^22^	*U*^33^	*U*^12^	*U*^13^	*U*^23^
O1	0.0992 (11)	0.0463 (6)	0.0503 (7)	0.0314 (7)	0.0388 (7)	0.0165 (5)
O2	0.0913 (11)	0.0402 (6)	0.0726 (8)	0.0205 (6)	0.0498 (8)	0.0096 (6)
O3	0.0476 (8)	0.0696 (8)	0.0573 (8)	0.0270 (6)	0.0158 (6)	0.0081 (6)
N1	0.0922 (13)	0.0422 (8)	0.0493 (8)	0.0227 (8)	0.0355 (8)	0.0142 (6)
N2	0.0491 (9)	0.0450 (7)	0.0508 (8)	0.0142 (6)	0.0180 (6)	0.0160 (6)
C1	0.0478 (10)	0.0381 (8)	0.0402 (8)	0.0191 (7)	0.0162 (7)	0.0144 (6)
C2	0.0769 (13)	0.0433 (9)	0.0463 (9)	0.0213 (9)	0.0281 (9)	0.0168 (8)
C3	0.0923 (16)	0.0427 (9)	0.0621 (11)	0.0231 (10)	0.0357 (11)	0.0241 (9)
C4	0.0978 (17)	0.0371 (9)	0.0669 (12)	0.0208 (10)	0.0369 (11)	0.0125 (9)
C5	0.132 (2)	0.0432 (10)	0.0587 (12)	0.0238 (11)	0.0485 (13)	0.0097 (9)
C6	0.0495 (10)	0.0409 (8)	0.0380 (8)	0.0218 (7)	0.0156 (7)	0.0135 (7)
C7	0.0499 (11)	0.0369 (8)	0.0492 (9)	0.0206 (7)	0.0211 (8)	0.0152 (7)
C8	0.0438 (9)	0.0325 (7)	0.0377 (8)	0.0152 (6)	0.0161 (7)	0.0093 (6)
C9	0.0419 (10)	0.0329 (7)	0.0491 (9)	0.0140 (7)	0.0199 (7)	0.0108 (7)
C10	0.0423 (9)	0.0346 (7)	0.0472 (8)	0.0126 (7)	0.0172 (7)	0.0075 (7)
C11	0.0404 (9)	0.0348 (7)	0.0496 (9)	0.0133 (7)	0.0132 (7)	0.0110 (7)
C12	0.0771 (14)	0.0387 (9)	0.0689 (12)	0.0200 (9)	0.0284 (11)	0.0131 (9)
C13	0.1020 (18)	0.0371 (10)	0.0973 (16)	0.0189 (11)	0.0391 (14)	0.0247 (11)
C14	0.0974 (18)	0.0550 (12)	0.0934 (16)	0.0188 (11)	0.0427 (14)	0.0405 (12)
C15	0.0765 (15)	0.0599 (11)	0.0652 (12)	0.0200 (10)	0.0317 (11)	0.0285 (10)
C16	0.0438 (9)	0.0261 (6)	0.0379 (7)	0.0128 (6)	0.0182 (6)	0.0112 (6)
C17	0.0494 (10)	0.0371 (7)	0.0351 (8)	0.0163 (7)	0.0183 (7)	0.0092 (6)
C18	0.0475 (10)	0.0432 (8)	0.0465 (9)	0.0205 (7)	0.0246 (8)	0.0102 (7)
C19	0.0417 (9)	0.0358 (7)	0.0475 (8)	0.0154 (7)	0.0174 (7)	0.0133 (7)
C20	0.0466 (10)	0.0410 (8)	0.0366 (8)	0.0135 (7)	0.0153 (7)	0.0071 (7)
C21	0.0464 (10)	0.0385 (8)	0.0405 (8)	0.0164 (7)	0.0222 (7)	0.0078 (6)

### Geometric parameters (Å, º)

**Table:**

O1—C6	1.2135 (17)	C8—H8A	0.996 (15)
O2—C10	1.2114 (18)	C9—C10	1.504 (2)
O3—C19	1.3689 (19)	C9—H9B	0.975 (18)
O3—H03A	0.93 (2)	C9—H9A	1.017 (17)
N1—C1	1.3295 (19)	C10—C11	1.504 (2)
N1—C5	1.338 (2)	C11—C12	1.387 (2)
N2—C15	1.339 (2)	C12—C13	1.372 (3)
N2—C11	1.340 (2)	C12—H12A	0.96 (2)
C1—C2	1.375 (2)	C13—C14	1.360 (3)
C1—C6	1.504 (2)	C13—H13A	0.99 (3)
C2—C3	1.375 (2)	C14—C15	1.376 (3)
C2—H2A	0.960 (18)	C14—H14A	0.95 (3)
C3—C4	1.357 (3)	C15—H15A	0.966 (19)
C3—H3A	0.94 (2)	C16—C21	1.389 (2)
C4—C5	1.368 (3)	C16—C17	1.391 (2)
C4—H4A	0.99 (2)	C17—C18	1.378 (2)
C5—H5A	0.99 (2)	C17—H17A	0.971 (16)
C6—C7	1.502 (2)	C18—C19	1.384 (2)
C7—C8	1.538 (2)	C18—H18A	0.938 (18)
C7—H7A	1.006 (19)	C19—C20	1.384 (2)
C7—H7B	1.001 (16)	C20—C21	1.381 (2)
C8—C16	1.513 (2)	C20—H20A	0.967 (17)
C8—C9	1.532 (2)	C21—H21A	0.998 (17)
			
C19—O3—H03A	109.1 (15)	H9B—C9—H9A	104.6 (13)
C1—N1—C5	116.28 (15)	O2—C10—C9	121.67 (14)
C15—N2—C11	116.90 (15)	O2—C10—C11	119.15 (13)
N1—C1—C2	122.88 (14)	C9—C10—C11	119.08 (13)
N1—C1—C6	117.47 (13)	N2—C11—C12	122.63 (15)
C2—C1—C6	119.64 (14)	N2—C11—C10	118.46 (13)
C1—C2—C3	119.43 (16)	C12—C11—C10	118.91 (15)
C1—C2—H2A	118.1 (11)	C13—C12—C11	119.0 (2)
C3—C2—H2A	122.4 (11)	C13—C12—H12A	121.8 (12)
C4—C3—C2	118.47 (17)	C11—C12—H12A	119.2 (12)
C4—C3—H3A	121.3 (12)	C14—C13—C12	118.97 (19)
C2—C3—H3A	120.2 (12)	C14—C13—H13A	121.0 (15)
C3—C4—C5	118.65 (17)	C12—C13—H13A	120.0 (15)
C3—C4—H4A	122.3 (12)	C13—C14—C15	119.1 (2)
C5—C4—H4A	119.1 (12)	C13—C14—H14A	123.3 (14)
N1—C5—C4	124.27 (18)	C15—C14—H14A	117.6 (15)
N1—C5—H5A	114.4 (13)	N2—C15—C14	123.4 (2)
C4—C5—H5A	121.3 (13)	N2—C15—H15A	115.5 (12)
O1—C6—C7	122.05 (14)	C14—C15—H15A	121.1 (12)
O1—C6—C1	118.79 (14)	C21—C16—C17	116.72 (14)
C7—C6—C1	119.14 (13)	C21—C16—C8	121.07 (13)
C6—C7—C8	112.24 (13)	C17—C16—C8	122.21 (13)
C6—C7—H7A	104.7 (10)	C18—C17—C16	121.93 (14)
C8—C7—H7A	109.3 (10)	C18—C17—H17A	121.3 (9)
C6—C7—H7B	110.5 (9)	C16—C17—H17A	116.7 (10)
C8—C7—H7B	111.5 (9)	C17—C18—C19	120.22 (15)
H7A—C7—H7B	108.3 (14)	C17—C18—H18A	120.7 (10)
C16—C8—C9	110.96 (12)	C19—C18—H18A	119.0 (11)
C16—C8—C7	110.90 (12)	O3—C19—C20	122.26 (14)
C9—C8—C7	110.75 (12)	O3—C19—C18	118.70 (14)
C16—C8—H8A	107.9 (9)	C20—C19—C18	119.04 (15)
C9—C8—H8A	108.1 (9)	C21—C20—C19	119.99 (14)
C7—C8—H8A	108.1 (8)	C21—C20—H20A	121.1 (10)
C10—C9—C8	112.42 (13)	C19—C20—H20A	118.9 (10)
C10—C9—H9B	104.4 (10)	C20—C21—C16	122.10 (14)
C8—C9—H9B	112.2 (10)	C20—C21—H21A	118.6 (9)
C10—C9—H9A	110.8 (9)	C16—C21—H21A	119.3 (10)
C8—C9—H9A	111.9 (10)		
			
C5—N1—C1—C2	0.8 (3)	C9—C10—C11—N2	11.1 (2)
C5—N1—C1—C6	−178.05 (18)	O2—C10—C11—C12	8.4 (3)
N1—C1—C2—C3	0.3 (3)	C9—C10—C11—C12	−168.07 (16)
C6—C1—C2—C3	179.14 (17)	N2—C11—C12—C13	−1.1 (3)
C1—C2—C3—C4	−0.7 (3)	C10—C11—C12—C13	178.02 (18)
C2—C3—C4—C5	0.1 (3)	C11—C12—C13—C14	0.1 (4)
C1—N1—C5—C4	−1.6 (4)	C12—C13—C14—C15	0.7 (4)
C3—C4—C5—N1	1.1 (4)	C11—N2—C15—C14	−0.4 (3)
N1—C1—C6—O1	164.80 (16)	C13—C14—C15—N2	−0.6 (4)
C2—C1—C6—O1	−14.1 (2)	C9—C8—C16—C21	64.71 (16)
N1—C1—C6—C7	−16.5 (2)	C7—C8—C16—C21	−58.83 (17)
C2—C1—C6—C7	164.56 (16)	C9—C8—C16—C17	−114.63 (14)
O1—C6—C7—C8	−30.0 (2)	C7—C8—C16—C17	121.83 (14)
C1—C6—C7—C8	151.38 (14)	C21—C16—C17—C18	0.2 (2)
C6—C7—C8—C16	−65.15 (17)	C8—C16—C17—C18	179.58 (13)
C6—C7—C8—C9	171.20 (13)	C16—C17—C18—C19	−0.3 (2)
C16—C8—C9—C10	72.76 (16)	C17—C18—C19—O3	−179.03 (13)
C7—C8—C9—C10	−163.62 (14)	C17—C18—C19—C20	0.2 (2)
C8—C9—C10—O2	16.4 (2)	O3—C19—C20—C21	179.21 (13)
C8—C9—C10—C11	−167.22 (13)	C18—C19—C20—C21	0.0 (2)
C15—N2—C11—C12	1.2 (3)	C19—C20—C21—C16	−0.1 (2)
C15—N2—C11—C10	−177.89 (16)	C17—C16—C21—C20	0.0 (2)
O2—C10—C11—N2	−172.47 (15)	C8—C16—C21—C20	−179.38 (13)

### Hydrogen-bond geometry (Å, º)

Cg3 is the centroid of the C16–C21 ring.

**Table:**

*D*—H···*A*	*D*—H	H···*A*	*D*···*A*	*D*—H···*A*
O3—H03*A*···N2^i^	0.93 (2)	2.00 (2)	2.8940 (19)	160 (2)
C12—H12*A*···O2^ii^	0.96 (2)	2.48 (2)	3.312 (3)	145 (2)
C4a—H4a···*Cg*3^iii^	0.99 (2)	0.98 (2)	3.825 (2)	144 (2)

Symmetry codes: (i) −*x*+1, −*y*+1, −*z*; (ii) −*x*+2, −*y*+2, −*z*+1; (iii) *x*, *y*−1, *z*.
